# Comparing access to sexual and reproductive health services among sexual minority youths and their peers: findings from a national survey in China

**DOI:** 10.1186/s12889-022-14508-8

**Published:** 2022-11-14

**Authors:** Yun Liang, Jiayi Hee, Chunxiao Peng, Chunyan Li, Wenzhen Cao, Kun Tang

**Affiliations:** 1 Vanke School of Public Health, Tsinghua University, 30 Shuangqing Road, Haidian District, Beijing, 100084 China; 2 School of Public Health, Peking University, Xueyuan Road 38, Beijing, 100191 China; 3 Department of Health Behavior, Gillings School of Global Public Health, University of North Carolina at Chapel Hill, Chapel Hill, North Carolina 27566 USA; 4 Tokyo College, The University of Tokyo, Bunkyo, Tokyo 113-8654 Japan; 5 School of Public Health and Management, Wenzhou Medical University, Wenzhou, 325035 China

**Keywords:** Sexual and reproductive health, Healthcare utilization, Sexual minority youths

## Abstract

**Background:**

Little is known about the access to measures of sexual and reproductive health (SRH) services among sexual minority communities in China, where sexuality-related stigma and discrimination remains high. The aim of this study is to investigate access to measures of SRH services among Chinese sexual minority youths (SMY) aged 17 to 24 years old.

**Methods:**

This cross-sectional study utilizes data on 54,580 youths from the 2019–2020 National College Student Survey on Sexual and Reproductive Health, conducted across 31 provinces in mainland China. Multivariable logistic regression modelling was utilized to assess the access to SRH services among Chinese youth with different self-reported sexual orientation.

**Results:**

The majority of respondents identified as heterosexual (77.6%). The remaining respondents identified as bisexual (9.0%), lesbian or gay (2.8%), others (3.02%), or unknown (7.51%). Gay men reported greater access to free contraceptives at health centers (OR 1.62, 95% CI: 1.32–1.99) and were more likely to have receive medical treatment for sexual and reproductive issues (OR 1.83, 95% CI: 1.26–2.63) compared to heterosexual men. Gay and bisexual men were also more likely to use condom at first sexual intercourse compared to heterosexual men (gay men: OR 1.38, 95% CI: 1.13–1.68; bisexual men: OR 1.33, 95% CI: 1.03–1.71). However, the associations were reversed among women (lesbians: OR 0.05, 95% CI: 0.03 to 0.08; bisexuals: 0.75, 95% CI: 0.65 to 0.86).

**Conclusions:**

Although SMY reported higher utilization of SRH services compared to their heterosexual counterparts, access to SRH services remains low among Chinese youths. Greater focus should be placed on improving access to SMY-friendly SRH services among Chinese youths.

## Background

Sexual minority youths (SMY) represent youths whose sexual orientation differs from commonly accepted societal or cultural norms. Studies have reported that SMY are more likely to experience poorer physical and psychological health outcomes compared to heterosexuals [1]. This can result in higher rates of substance abuse, sexually transmitted infections (STIs), cancer, obesity, depression, self-harm, and suicide [2–9]. This is because as posited by the minority stress model, there exists prejudices and hostility towards SMY. Therefore, SMY are more likely to conceal their sexual minority identity and internalized homophobia that invokes feelings of exclusion, rejection, and discrimination [10]. Adverse outcomes are further exacerbated by the avoidance of seeking medical support or assistance in fear of discrimination and stigma in healthcare settings.

Published studies investigating access to measures of sexual and reproductive health (SRH) services among SMY mainly focuses on HIV-related health services, sexual behaviors [11, 12] and mental health services [13, 14]. Few studies investigate the access to general SRH services. A study indicated that SMY were more likely to search online for medical information compared to heterosexuals [15]. Studies have also reported that lesbian and bisexual women often face greater difficulties in receiving medical services than heterosexual women [16]. As lesbian and bisexual women are more likely to experience a range of barriers including prejudicial conduct by healthcare professionals, institutional homophobia, fear of disclosure of sexual orientation status, and exposure to negative reactions [17], this may pose difficulties when seeking medical advice for concerns such as practicing safer sex with same-sex partners, that are unique to lesbians and bisexual individuals [18]. Lesbians, gay men, and bisexual individuals are also less likely to have access to care in regular healthcare [19] and are less likely to disclose their sexualities to healthcare providers [20]. Yet, studies have reported that SMY are more likely to seek mental health support and services and have greater access to mental health services than heterosexuals [21].

Whilst there is an increase of emphasis on sexual equality as promoted by the World Health Organization (WHO), there still exist high levels of sexual inequality in China [22]. SRH services, particularly for SMY, needs to be contextualized to Chinese society and culture. Homosexuality was previously classified as a sexual disorder in the Chinese Classification of Mental Disorders (CCMD-1) in 1978 [23], and was also previously associated with hooliganism as stated in the Chinese Criminal Law in 1979 [24]. At present, marriage between same-sex couples has yet to be legalized in China and therefore, same-sex couples are not able to access the same rights and protection (e.g., marriage and adoption of children) as heterosexual couples [25]. Given the sensitive nature of discussing sexual health in China, particularly in unmarried young adults, public health programs are few. However, public health interventions to provide comprehensive and standardized sexuality education conducted in rural regions of China have been performed and have reported promising results one-year post-intervention [26]. Furthermore, with the high prevalence of HIV among certain populations, the Chinese government have increased the focus on men who have sex with men (MSM) and transgenders (TG) in recent years, by providing interventions and programs to enhance health services and sexual education [27].

Access to appropriate SRH services is important given that China has the largest lesbian, gay, bisexual, and transgender (LGBT) population [28]. However, studies that assesses accessibility to SRH services among SMY are lacking. Therefore, to address the gap in the present literature, our study aims to investigate the access to measures of SRH services among SMY in China, for the development of effective interventions and support systems. Increased access to SRH services among Chinese youths with various sexual orientation is key in the WHO Sustainable Development Goal for achieving health and well-being for all, and sexual equality.

## Methods

### Respondents and procedure

Data on 54,580 Chinese college students from the National College Student Survey on Sexual and Reproductive Health 2019–2020 (NCSS-SRH) was utilized. The NCSS-SRH was conducted by the China Family Planning Association (CFPA) and collected by the China Youth Network (CYN). An internet-based self-administered questionnaire was distributed using between November 2019 and February 2020 to a total of 241 institutions of higher education, including key universities (double first-class universities), ordinary universities, and colleges, after balancing for the type of university and vocational college. The questionnaire was delivered to the students through contact points from the selected educational institutions using convenience sampling. The methods of survey instrument development and study design has been previously described in detail elsewhere [29].

A total of 55,757 responses from 1,764 universities and vocational colleges were collected. Responses were excluded if the respondent (1) did not provide informed consent, (2) answer all questions, (3) pass consistency checks and logic verification (checks that ensures the correct behavior is demonstrated by respondents based on specifications), (4) were not between the ages of 17–24, (5) and/or were not enrolled as a full-time student at a Chinese university. The remaining 54,580 responses were included in final analyses. This study has been reviewed and approved by the Institutional Review Board of Tsinghua University (IRB No. 20190083).

### Measures

#### Access to SRH services

Access to SRH services were measured using 3 questions: “Have you ever received free contraceptives from public health institutions or clinics such as the local centers for disease prevention and control (CDC), community health centers?”, “Did you use a condom during your first sexual intercourse (penetrative sex)?”, and “Have you ever receive any medical treatment for reproductive health problems in the past 12 months?”. Self-reported reproductive health problems include urethral or vaginal discharge, painful urination, genital inflammation, genital ulcers, genital herpes, genital itching, and hematuria or vaginal bleeding. Response options were “yes”, coded as “1″ or “no”, coded as “0″. Respondents who answered “Yes” to receiving medical treatment for reproductive health problems in the past 12 months were then asked the following question: ‘‘If you have had the symptoms mentioned above, which of the following medical institutions did you receive treatment at?” Response options were ‘‘never diagnosed or treated”, “public health institution (including public hospital)”, “private hospital”, “private clinic”, “self-administered medication”, and “others”. Responses “public health institution (including public hospital)”, “private hospital”, “private clinic”, “self-administered medication”, and “others” were classified as having received medical treatment, while “never diagnosed or treated” was classified as not having received medical treatment.

#### Sexual orientation

Sexual orientation was measured using the question ‘‘Which one do you think best describes your sexual orientation?’’. Response options were “heterosexual”, “same-sex attraction”, “bisexual”, “pansexual”, “asexual”, “others”, “unknown” or “uncertain”. Responses “pansexual”, “asexual”, and “others” were classified as others, while the remaining responses were analyzed as established.

#### Sociodemographic variables

Data on age, ethnicity, educational level, parents’ highest educational qualifications, hometown region, region of university or college, only-child status, family economic status, left-behind experience (defined as remaining in their hometown while one or both parents relocate interstate for work), migration history, sexual behavior, and SRH in the past 12 months were collected. Ethnicity was classified into Han or ethnic minorities. Hometown region, defined as the place of residence before attending university or vocational college, was classified into urban, suburban, and rural (as reflected on the national identification document of each individual). Only-child status was classified as only-child or students with siblings. Family economic status was assessed using the question: “How would you rate your family financial situation?” Response options ranged from “1 (very poor)” to “7 (very good)”. Scores were classified into inferior (less than 4), moderate (4 to 5), and superior (greater than 5).

#### Statistical analysis

Continuous variables were described as mean ± standard deviation, while categorical variables were described as proportion. Multivariable logistic regression was utilized to investigate the association between different sexual orientations, demographic, and region of university or college with access to SRH services. The models were adjusted for sex, age, ethnicity, education level, parents’ highest educational qualifications, hometown region, only-child status, family economic status, left-behind experience, and migration history. Results were expressed as odds ratios (ORs) and 95% confidence intervals (95% CIs). The level of statistical significance was set at 5% (*p* < 0.05) for all statistical analyses. All data analyses were performed using Stata/SE version 16.0 (Stata Corp, Texas, USA).

## Results

### Characteristics of survey respondents

The characteristics of respondents according to sex are presented in Table 1. On average, respondents were 199 ± 1.8 years old. 77.6% of respondents self-identified as heterosexuals (82.8% males and 74.9% females), while the remaining 9.0% self-identified as bisexuals (5.0% males and 11.1% females), 2.8% as lesbian or gay men (5.4% males and 1.5% females), 3.02% as others (2.17% males and 3.48% females), and 7.51% as unknown (4.64% males and 9.03% females). The majority of the respondents were of Han ethnicity (90.6%), were junior college and undergraduate students (96.6%), had parents with education levels middle school and below (78.5% fathers and 82.6% mothers), resided in suburban or rural regions (55.8%), had siblings (68.5%), were moderately financially well-off (11.0%), and had left behind (31.3%) and migration (20.0%) experiences. 22.5% of respondents reported having had sexual intercourse, while 19.8% reported having had reproductive health problems in the past 12 months.

**Table 1:** Characteristics of respondents (*N* = 54,580)

Characteristics	Male (*n* = 18,844)	Female (*n* = 35,736)	Total (*N* = 54,580)	*P*-value
**Age (years), mean/SD**	19.94/1.95	19.86/1.75	19.88/1.82	< 0.0001
**Sexual orientation, % (n)**				
Heterosexual	82.81 (15,605)	74.90 (26,767)	77.63 (42,372)	< 0.0001
Homosexual	5.35 (1,008)	1.46 (520)	2.80 (1,528)	
Bisexual	5.03 (948)	11.14 (3,980)	9.03 (4,928)	
Other	2.17 (408)	3.48 (1,243)	3.02 (1,651)	
Uncertain	4.64 (875)	9.03 (3,226)	7.51 (4,101)	
**Ethnicity**				
Han	91.71 (17,282)	90.07 (32,186)	90.63 (49,468)	< 0.0001
Ethnic minorities	8.29 (1,562)	9.93 (3,550)	9.37 (5,112)	
**Educational level**				
College	40.25 (7,585)	32.65 (11,668)	35.27 (19,253)	< 0.0001
University	55.70 (10,497)	64.22 (22,951)	61.28 (33,448)	
Graduate	4.04 (762)	3.13 (1,117)	3.44 (1,879)	
**Father’s highest educational level**				
Primary school and below	18.78 (3,538)	19.50 (6,968)	19.25 (10,506)	0.042
Middle school	59.31 (11,177)	59.27 (21,181)	59.29 (32,358)	
College degree or above	19.93 (3,755)	19.48 (6,960)	19.63 (10,715)	
Uncertain	1.98 (374)	1.75 (627)	1.83 (1,001)	
**Mother’s highest educational level**				
Primary school or below	29.92 (5,638)	30.59 (10,933)	30.36 (16,571)	< 0.0001
Middle school	52.28 (9,852)	52.24 (18,669)	52.26 (28,521)	
College degree or above	15.56 (2,933)	15.61 (5,579)	15.6 (8,512)	
Uncertain	2.23 (421)	1.55 (555)	1.79 (976)	
**Hometown region**				
Urban	45.08 (8,494)	43.69 (15,614)	44.17 (24,108)	< 0.0001
Suburban	32.68 (6,159)	34.37 (12,281)	33.79 (18,440)	
Rural	22.24 (4,191)	21.94 (7,841)	22.04 (12,032)	
**Only-child status**	38.51 (6,960)	28.08 (9,791)	31.64 (16,751)	< 0.0001
**Family economic status**				
Inferior	26.39 (4,973)	24.85 (8,882)	25.38 (13,855)	< 0.0001
Moderate	61.63 (11,614)	64.66 (23,107)	63.61 (34,721)	
Superior	11.98 (2,257)	10.49 (3,747)	11.00 (6,004)	
**Left-behind experience (Yes)**	30.73 (5,790)	31.59 (11,290)	31.29 (17,080)	0.038
**History of migration (Yes)**	21.33 (4,019)	19.24 (6,875)	19.96 (10,894)	< 0.0001
**Ever had sexual intercourse (Yes)**	28.19 (5,312)	19.50 (6,968)	22.5 (12,280)	< 0.0001
**Had symptoms related to SRH in the past 12 months (Yes)**	12.79 (2,410)	23.44 (8,376)	19.77 (10,786)	< 0.0001

Values in the table are presented as percentages (%) and proportion unless specified otherwise

*SD* Standard deviation, *SRH* Sexual and reproductive health

### Access to SRH services by sexual orientation

The associations between sexual orientation among respondents who have had sexual intercourse or reproductive health problems in the past 12 months with access to SRH services are presented in Table 2. 19.0% of heterosexual men, 9.4% of heterosexual women, 9.3% of lesbians, 26.8% of gay men, 22.5% of bisexual men and 10.8% of bisexual women reported having received free contraceptives from health services, while 62.9% of heterosexual men, 66.1% of heterosexual women, 10.7% of lesbians, 70.8% of gay men, 71.6% of bisexual men and 62.0% of bisexual women reported condom use at first sexual intercourse.

**Table 2:** Accessibility to sexual and reproductive health services among college students with different self-identified sexual orientations

**Variable**	**Male**					**Female**					**Total**
	**Heterosexual**	**Homosexual**	**Bisexual**	**Others**	**Uncertain**	**Heterosexual**	**Homosexual**	**Bisexual**	**Others**	**Uncertain**	
**Accessibility to sexual and reproductive health services among individuals who have had sexual intercourse (*N* = 12,280)**											
Have received free contraceptives from formal health services	19.00 (17.84 to 20.22)	26.79 (23.43 to 30.43)	22.49 (18.33 to 27.26)	20.00 (12.90 to 29.68)	17.98 (11.24 to 27.50)	9.36 (8.59 to 10.19)	9.27 (5.97 to 14.11)	10.81 (9.19 to 12.68)	17.35 (12.87 to 22.98)	9.72 (6.41 to 14.48)	14.31 (13.70 to 14.94)
Condom use at first sexual intercourse	62.86 (61.39 to 64.32)	70.78 (67.06 to 74.24)	71.60 (66.54 to 76.17)	56.67 (46.15 to 66.62)	59.55 (48.94 to 69.34)	66.05 (64.73 to 67.33)	10.73 (7.15 to 15.80)	62.03 (59.28 to 64.71)	56.62 (49.94 to 63.07)	62.50 (55.82 to 68.74)	63.68 (62.83 to 64.53)
**Accessibility to sexual and reproductive health services among individuals who have had symptoms related to reproductive health problems in the past 12 months (*N* = 10,788)**											
Have received medical treatment	26.32 (24.42 to 28.31)	41.06 (33.44 to 49.13)	31.79 (24.81 to 39.69)	19.57 (10.32 to 33.96)	36.67 (27.27 to 47.21)	35.39 (34.20 to 36.60)	29.69 (22.36 to 38.24)	40.41 (37.63 to 43.26)	41.55 (35.93 to 47.40)	30.55 (27.14 to 34.18)	34.05 (33.16 to 34.95)
Public health institution (including public hospital)	17.19 (15.59 to 18.92)	21.19 (15.36 to 28.50)	19.21 (13.65 to 26.34)	15.22 (7.28 to 29.09)	22.22 (14.72 to 32.10)	24.77 (23.71 to 25.87)	21.09 (14.83 to 29.10)	29.37 (26.82 to 32.05)	29.58 (24.54 to 35.17)	20.97 (18.03 to 24.26)	23.54 (22.75 to 24.35)
Private hospitals	2.74 (2.10 to 3.56)	4.64 (2.21 to 9.46)	3.97 (1.78 to 8.61)	4.35 (1.05 to 16.32)	5.56 (2.30 to 12.80)	2.38 (2.03 to 2.79)	1.56 (0.39 to 6.11)	2.14 (1.45 to 3.15)	2.46 (1.18 to 5.09)	2.13 (1.26 to 3.56)	2.48 (2.21 to 2.80)
Private clinics	2.03 (1.49 to 2.75)	1.32 (0.33 to 5.19)	1.99 (0.64 to 6.03)	2.17 (0.29 to 14.55)	6.67 (2.99 to 14.19)	2.08 (1.76 to 2.47)	0.00 (-)	1.20 (0.71 to 2.01)	1.41 (0.53 to 3.71)	1.52 (0.82 to 2.80)	1.93 (1.68 to 2.21)
Self-administered medication	7.00 (5.95 to 8.21)	17.22 (11.96 to 24.15)	11.26 (7.09 to 17.42)	4.35 (1.05 to 16.32)	8.89 (4.46 to 16.92)	10.31 (9.57 to 11.10)	7.81 (4.23 to 13.99)	11.64 (9.93 to 13.62)	11.97 (8.67 to 16.31)	9.12 (7.14 to 11.57)	9.86 (9.31 to 10.44)

Values in the table are percentages (%) and 95% confidence intervals (95% CIs) unless otherwise specified

Others, Pan sexual, Asexuality, others etc.; SRH

34.1% of respondents who reported reproductive health problems in the past 12 months have received medical treatment; this rate was higher in heterosexual women compared to heterosexual men (35.4% vs. 26.3%), and in bisexual women compared to bisexual men (40.4% vs. 31.8%). However, the rate was lower in lesbians compared to gay men (29.7% vs. 41.1%). The majority of respondents reported receiving medical treatments from public health institutions (23.5%).

### Differences in access to SRH services

The associations between sex and access to SRH services, stratified by sexual orientation is presented in Table 3. Gay men were significantly more likely to have received free contraceptives from health services (OR 1.62, 95% CI: 1.32–1.99), and to receive medical treatment for reproductive health problems over the past 12 months (OR 1.83, 95% CI: 1.26–2.63), compared to heterosexual men. Gay men and bisexual men were also significantly more likely to have used a condom at first sexual intercourse (gay men: OR 1.38, 95% CI: 1.13–1.68; bisexual men: OR 1.33, 95% CI: 1.03–1.71), compared to heterosexual men.

**Table 3:** Relative odds of accessibility to SRH services among college students with different sexual orientation

**Variable**	**Male**					**Female**				
	**Heterosexual**	**Homosexual**	**Bisexual**	**Others**	**Uncertain**	**Heterosexual**	**Homosexual**	**Bisexual**	**Others**	**Uncertain**
Have received free contraceptives from formal health services	1.00(Ref)	1.62* (1.32 to 1.99)	1.22 (0.93 to 1.61)	1.12 (0.65 to 1.92)	0.88 (0.50 to 1.54)	1.00(Ref)	0.96 (0.59 to 1.56)	1.14 (0.92 to 1.40)	1.92* (1.32 to 2.78)	0.88 (0.53 to 1.44)
Condom use at first sexual intercourse	1.00(Ref)	1.38* (1.13 to 1.68)	1.33* (1.03 to 1.71)	0.68 (0.44 to 1.06)	0.93 (0.60 to 1.45)	1.00(Ref)	0.05* (0.03 to 0.08)	0.75* (0.65 to 0.86)	0.57* (0.43 to 0.76)	0.81 (0.61 to 1.09)
Have received medical treatment	1.00(Ref)	1.83* (1.26 to 2.63)	1.36 (0.94 to 1.97)	0.57 (0.26 to 1.24)	1.64* (1.03 to 2.61)	1.00(Ref)	0.69 (0.47 to 1.02)	1.14 (1.00 to 1.30)	1.18 (0.92 to 1.51)	0.82* (0.68 to 0.98)

Values in the table are odds ratios (ORs) and 95% confidence intervals (95% CIs). “*” means that *P* value is less than 0.05

Data were adjusted for age, ethnic, education level, fathers’ and mothers’ education level, hometown region, family structure (one-child family or not), family economic status, history of left behind, history of migration

However, the associations were reverse in women. Lesbians and bisexual women were less likely to have used a condom at first sexual intercourse, (lesbians: OR 0.05, 95% CI: 0.03–0.08; bisexuals: OR 0.75, 95% CI: 0.65–0.86), compared to heterosexual women. The association between lesbians and receiving free contraceptives from health services, as well as receiving medical treatment for reproductive health problems over the past 12 months compared to heterosexual women were not statistically significant.

## Discussion

Our study findings demonstrated that gay men were more likely to have access to free contraceptives and were more likely to seek medical treatment for reproductive health problems as compared to their heterosexual counterparts. Condom use during first sexual intercourse was also higher among bisexual and gay men. However, among women, particularly lesbians and bisexuals, the association between sexual orientation and condom use during first sexual intercourse was reversed. No significant differences were found between women with different sexual orientations with regards to their actual access to SRH services.

We found that gay men had the highest rate of access to free contraceptives and were more likely to seek medical treatment for reproductive health problems compared to heterosexual men, consistent with findings from the 2016 national survey on SMY in China, which reported that a lower proportion of SMY encountered difficulties when receiving health care services [30]. However, previous studies have reported that SMY were more likely to have less access to SRH services, resulting in low health service utilization [31]. Concerns with privacy and confidentiality may also deter SMYs from discussing their sexuality with healthcare providers [32]. As the number of new HIV infections among Chinese college students has increased significantly (annual growth rate range from 30 to 50%) [33], the Chinese government announced a policy in 2015 to increase HIV health care services in colleges. This included the provision of anonymous HIV urine-testing services in over 40 universities [34]. Past efforts to enhance HIV/AIDS-related knowledge among MSM in Chinese colleges may have increased awareness of safer male-male sex [35, 36], contributing to higher SRH awareness and higher SRH services utilization in gay men, explaining our findings.

Although the findings of this study suggests that SMY have better SRH services utilization than heterosexuals, the absolute rate of SRH services utilization remains low, suggesting that the majority of SMY and their heterosexual peers do not have access to appropriate SRH services. This is consistent with the findings of another study, which reported that the proportion of college students who received reproductive health services remains low due to high medical costs and indifferent attitudes of health providers [37]. Therefore, it is important to increase access to student friendly SRH services, reduce medical costs, and provide training and comprehensive sexuality education to health providers to decrease discrimination in clinical settings.

We also found that bisexual men and gay men had the highest rates of condom use during first sexual intercourse compared to men with other sexual orientations. However, a study conducted by Liu et al*.* (2015) in China reported that gay and bisexual men were less likely to use a condom during sexual intercourse [38]. A possible explanation for this difference in findings could be because our study assessed the status of condom use at the first sexual intercourse, while the study by Liu et al*.* assessed the status of condom use for most episodes of sexual intercourse. This may suggest that while sexual minority males may have higher awareness of using contraception at first sexual intercourse, this awareness declines in subsequent sexual intercourses, although there is no evidence in our study to support this hypothesis. However, the study by Liu et al. (2015) and other studies demonstrated consistent results to our findings that lesbians and bisexual women were less likely to use condoms at first sexual intercourse compared with heterosexual women [39–41]. This could be because female-female sexual intercourse is assumed to be safer than heterosexual or male-male sexual intercourse, or because condoms is not needed. Given the barriers and difficulties faced by lesbians [42], exploring their needs is an important area for future research.

Overall, this study has several limitations. First, because the internet-based questionnaire utilizes self-reporting and because sexual-related topics remains a taboo subject in China, response bias may be present. However, we attempted to mitigate this bias by utilizing an internet-based questionnaire, as well as by conducting thorough logic checks before data analysis. Second, although our intentions were to use variables such as accessibility to condoms at first sex, due to the limitations of survey measurements, we were only able to examine whether condoms had been used during first sex. Third, as same-sex attraction was used as a proxy sexual identification, this is likely to result in an overestimation of results given that same-sex attraction may be subjected to individual interpretation and loosely interpreted as, for example, attraction to a celebrity of the same-sex. Fourth, given that this study was conducted retrospectively, recall bias may be present. Last, because the study utilized a cross-sectional study design, causality cannot be inferred.

However, this study also has several strengths. Over the last decade, with the exception of family planning and HIV or acquired immunodeficiency syndrome prevention and control, SRH has received little attention in policy or public health research in China. Utilizing the largest and most recent national survey on the SRH of college students in China, our study is the most comprehensive study to investigate the access to SRH services for youths with various sexual orientation in China using three major indicators of SRH; access to free condoms at public health institutions, condom use at first sexual intercourse, and access to medical treatment for reproductive health problems in the past 12 months. Our findings can help provide evidence-based information to inform educational institutions of SRH topics that should be prioritized, as well as help inform community clinics to increase their provision of inclusive SRH services. Given that our study is the largest study performed on SRH in Chinese college students from various areas across China, a large diversity of students has been covered allowing for better understanding of SRH in Chinese college students.

## Conclusions

While our results indicate that SMY better utilize SRH services compared to heterosexuals, the absolute rates of accessibility to SRH services remains low in Chinese youths. Increased access to SRH services can help reduce sexually transmitted infections and eliminate the stigma around sexual health in China. Future policies should pay greater attention to the accessibility of SRH services among both SMY and heterosexuals.

## Data Availability

The datasets used and analysed during the current study are available from the corresponding author on reasonable request.

## Abbreviations

SRH: Sexual and Reproductive Health
SMY: Sexual Minority Youth
NCSS-SRH: National College Student Survey on Sexual and Reproductive Health
CFPA: China Family Planning Association
CYN: China Youth Network
CDC: Centers for Disease Prevention and Control
OR: Odds Ratio
HIV: Human Immunodeficiency Virus
AIDS: Acquired Immunodeficiency Syndrome
MSM: Men who have sex with men
SGM: Sexual and gender minority
